# Challenges in treatment of posttraumatic stress disorder in refugees: towards integration of evidence-based treatments with contextual and culture-sensitive perspectives

**DOI:** 10.3402/ejpt.v6.24750

**Published:** 2015-01-07

**Authors:** Boris Drožđek

**Affiliations:** Psychotrauma Centrum Zuid Nederland, Reinier van Arkel groep, Den Bosch, The Netherlands

**Keywords:** refugees, PTSD, intervention, therapy, context, Cultural Formulation Interview, organized violence, culture

## Abstract

**Background:**

Research shows that trauma-focused therapy and multimodal interventions are the two most often used strategies in treatment of refugees suffering from posttraumatic stress disorder (PTSD). While preliminary evidence suggests that trauma-focused approaches may have some efficacy, this could not be established for multimodal interventions. However, it may be that multimodal interventions have been studied in more treatment-resistant refugees with very high levels of psychopathology, disability, and chronicity. In the past decades, various models for understanding of the complex relationship between mental health problems and well-being have emerged. They aim at framing mental health problems in individualized, contextual, epigenetic, and culturally sensitive ways, and may be useful in tailoring content and timing of multimodal interventions.

**Objective:**

To draw clinicians’ attention to the possibility of using the Integrative Contextual Model for understanding and assessment of posttrauma mental health sequelae while tailoring multimodal interventions; to present a possible way of combining multimodal with evidence-based trauma-focused approaches; and to improve the understanding and treatment of PTSD and other mental health problems in refugee survivors of prolonged and repeated trauma.

**Method:**

Based on literature, clinical experience, and presentation of a fictional case, the use of the Integrative Contextual Model in tailoring the treatment of severe PTSD in a refugee patient is presented and discussed.

**Results:**

The Integrative Contextual Model for understanding and assessing factors, which may play a role in causing and maintaining of PTSD and comorbidity in refugees, may help tailoring of multimodal interventions. These interventions can be combined with evidence-based trauma-focused treatments.

**Conclusion:**

The field of refugee mental health intervention and clinical practice with traumatized refugees may be enriched with the use of contextual and developmental models for assessment and conceptualization of posttrauma sequelae. Multimodal and trauma-focused interventions may be applied sequentially in a course of the treatment trajectory.

In 2008, the International Society for Traumatic Stress Studies (ISTSS) published the last review of the international evidence-based guidelines for the treatment of posttraumatic stress disorder (PTSD) (Foa, Keane, Friedman, & Cohen, 2008). Since these guidelines did not include recommendations regarding the treatment of survivors of prolonged and repeated trauma, the ISTSS expert group has, in 2012, formulated the additional consensus treatment guidelines for complex PTSD in adults (Cloitre et al., 2012). With complex PTSD is meant severe PTSD with comorbidity (e.g., dissociation, depression, substance abuse, personality disorders), as complex PTSD is not recognized as a discrete disorder in psychiatric classifications (Resick et al., 2012).

The 2008 evidence-based guidelines recommend trauma-focused cognitive-behavioral treatment (CBT) as the first choice intervention in the treatment of PTSD. Moreover, other recent meta-analytic reviews (Diehle, Schmitt, Daams, Boer, & Lindauer, 2014; Powers, Halpern, Ferenschak, Gillihan, & Foa, 2010) found that prolonged exposure (imaginal and *in vivo*) alone or in combination with cognitive restructuring show the largest effects in PTSD treatment. Prolonged exposure can be also effectively used in patients with PTSD and comorbidity, and research (Van Minnen, Harned, Zoellner, & Mills, 2012) suggests that its use is often associated with a decrease of both PTSD and comorbid problems. However, in cases with severe comorbidity, prolonged exposure should be integrated with treatment of comorbid conditions.

With regard to severe PTSD with comorbidity (complex PTSD) in survivors of prolonged and repeated trauma, the 2012 consensus guidelines recommend a sequential, phase-based treatment model based on a hierarchy of survivors’ treatment needs assessed prior to treatment. The first phase should focus on stabilization and the strengthening of daily life skills. It includes interventions aiming at improving patients’ safety and resources, developing capacities for emotional awareness and expression, and increasing interpersonal and social competencies. In the second phase, the review and reappraisal of trauma memories in an organized way should take place. In this phase, the evidence-based trauma treatments such as CBT (Hinton, Rivera, Hofmann, Barlow, & Otto, 2012), eye movement desensitization and reprocessing (EMDR) (Shapiro, 2001), or narrative exposure therapy (NET) (Morina et al., 2012) can be applied. Phase 3 should involve consolidation of treatment gains and focus at enabling patients’ transition from the treatment to a life in the community.

In the population of refugees, the impacts of trauma can be severe. A critical review of psychological treatments of PTSD (Nickerson, Bryant, Silove, & Steel, 2011) in this population pointed out that refugees with PTSD are, at first, exposed to pre-migration traumatic events, and then, later on, have to deal with resettlement difficulties, and the postmigration stressors, such as immigration detention and temporary protection with a threat of repatriation. In the field of refugee mental health intervention, two contrasting approaches with regard to treatment of PTSD are applied—trauma-focused therapy and multimodal interventions. While trauma-focused interventions aim at discussing traumatic experiences and targeting PTSD symptoms, multimodal treatments address concurrently a variety of issues, like ongoing psychosocial difficulties, psychological functioning, physical health, and acculturation problems. Preliminary research evidence (Nickerson et al., 2011) suggests that trauma-focused approach may have some efficacy in treating PTSD and associated distress in refugees, while research has failed to establish the same for multimodal treatments. However, multimodal approaches are still frequently applied in daily practice, and it is possible that they fail to reduce psychopathology because of high levels of symptom severity, disability, and chronicity in refugees seeking help at specialized refugee treatment centers. More recent research (Drožđek, Kamperman, Bolwerk, Tol, & Kleber, 2012; Drožđek, Kamperman, Tol, Knipscheer, & Kleber, 2014) suggests that a combination of multimodal and trauma-focused group treatments in a specialized refugee trauma treatment setting may reduce psychopathology in refugees both on the short term and on longer term.

The aim of this article is to help clinicians tailor multimodal interventions and combine them with trauma-focused interventions in the treatment of refugee patients with PTSD. This combination of interventions may be useful in cases where a refugee is either unwilling to confront traumatic memories right away from the start of treatment or is severely disturbed due to suffering from PTSD with multiple comorbid conditions and current psychosocial stress. Also, combining interventions may be needed in case a refugee has explanatory models for his current condition which is different from the one rooted in western culture and science. A model for understanding the complex array of psychological reactions, which may occur in refugees with severe PTSD and comorbidity, will be presented, and its use will be illustrated in a case presentation. Our aim is to provide additional perspectives for a better inclusion of individual patient's preferences and values, and contextual factors which may influence mental health problems, and to enrich the evidence-based practice with the refugee survivors of prolonged and repeated trauma.

## Models for contextual conceptualization of impacts of trauma and the “refugee experience”

In the past decennia, different models aiming at being inclusive with regard to issues of diversity and contextual determinants of psychopathological phenomena have been developed. They originate from the fields of systems theory, cultural psychiatry, migrant mental health, and posttraumatic stress theory. They may help understanding multiple impacts of exposure to traumatic experiences in refugees in an adequate way.

The concept of cumulative trauma (Khan, 1977) sees trauma as a product of a series of non-traumatic experiences which may accumulate over time within the interaction of an individual with its environment, and may finally lead to emergence of mental health problems. Khan (1977) introduced the dimensions of time and the interactive relationship between an individual and its surroundings into the discussion about trauma, and converted the event (traumatic experience) into a process (Becker, 2004). Building on Khan's work, Kielson (1992) introduced the concept of sequential traumatization. He suggested that there is no “post” in PTSD, but that the ever changing environmental/historical context of the individual survivor interacts with a traumatic experience through time, allowing the quality and the quantity of the traumatic sequences to be different in various contexts and at different times across the lifespan (Becker, 2004). These concepts are in line with the more recent ideas considering the development of severe PTSD with comorbid conditions and suggesting that complex posttraumatic symptomatology may be characterized as a dimension of symptomatic severity in a continuum of pathological reactions to trauma (Resick et al., 2012).

In order to explore and understand the interaction of an individual with its environment, Bronfenbrenner (1981) developed the Ecological Systems Theory. He described “the ecological environment” as an arrangement of different levels/dimensions wherein an individual is nested. These are seen as concentric structures, each contained within the next. These structures are referred to as the micro- (intrapsychic), meso- (peer group, family, social life), exo- (society), and macrosystems ((sub) culture, belief systems, ideology). The theory looks at individual development within the context of the system of relationships that form the environment. Influences, within and between the systems, are suggested to be bidirectional, and changes or conflicts in any one dimension of the system ripple throughout other dimensions. According to this theory, problems that psychiatric patients are presenting can be defined as psychological or intrapsychic, but yet at the same time as medical, social, political, cultural, existential, or as multidimensional. A mental health provider should, therefore, transcend the either/or attitude, and search for a broader conceptual frame. This will enable exploration of the interplay of internal and external influences hindering or facilitating a patient's individual development.

Hobfoll's (1998, 2001) Conservation of Resources (COR) theory views resource loss as the key component in the process of development of mental health problems. This theory is based on the tenet that individuals strive to obtain, retain, protect, and foster things that they value. The stress process is placed in the context of a general model of social action, and stress arises in situations where individuals face a threat of resource loss, where resources are actually lost, or where an investment of resources fails to produce an expected return. COR theory provides the tools to examine objective conditions out of which stressful demands are born, and is seen as an alternative to appraisal-based stress theories. According to the COR theory, individuals accumulate resources in order to accommodate, withstand, or overcome threats. They may accumulate personal resources, such as self-esteem, material resources, such as money, and condition resources, such as status, and social support. Stressful or traumatic events consume these resources, thereby augmenting one's sensitivity to subsequent stressors. COR theory analyses the flux of resources at times of stress.

**Fig. 1:**
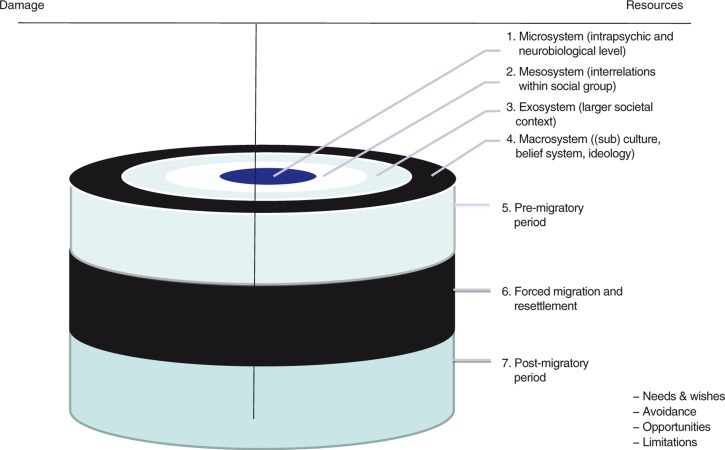
The Integrative Contextual Model.

Building on the above-mentioned theories, Drožđek (2007) and Drožđek, Wilson, and Turkovic (2012a, 2012b) have formulated the Integrative Contextual Model for understanding, and assessment of posttrauma mental health sequelae. This model brings together the developmental and the ecological perspectives (Fig. 1). It points out that, in assessing and treating mental health problems, a clinician should acknowledge the necessity of simultaneously focusing on the intrapsychic and biological dimensions, and on the interpersonal and socio-political dimensions of human experiences. This model underlines the importance of understanding the context of an individual as a dynamic system that can change over time. Moreover, it points out that the relationship between mental health disorders and a context is bidirectional. A psychiatric diagnosis is viewed as a current reflection of a psychological imbalance, a mirror of one's lifelong dynamic struggle between sources of resilience and damage. A mental health professional is challenged to identify and evaluate the risk and the protective factors in both the developmental and the socio-environmental contexts of a patient, recognizing, thereby, the influence of culture on human psychology. In other words, the model suggests that assessing mental health disorders is like watching a movie of one's life in order to understand a person with mental health problems, and not just taking his/her snapshot.

The recent, fifth edition of the Diagnostic and statistical manual of mental disorders (DSM) (American Psychiatric Association [APA], 2013) acknowledges the importance of context and culture in psychiatric practice more than the previous ones. It contains the Cultural Formulation Interview (CFI). CFI is based on the Cultural Formulation of Diagnosis (CFD) (Lewis-Fernandez, 1996), which is an integrative formulation that explores a wide range of cultural and social issues relevant for understanding mental health problems in individuals across the world, and helps tailoring appropriate treatment options. We consider CFI to be a very useful tool for understanding impacts of trauma on lives and mental health of refugees.

Next, we will illustrate how the Integrative Contextual Model (Drožđek, 2007; Drožđek et al., 2012a, 2012b) can be applied in a refugee patient in order to understand and assess the impacts of contextual factors on causes, maintenance, and treatment of PTSD, and help tailoring treatment strategies.

## Case presentation

Joseph, a 37-year-old man from Liberia left his home country alone 17 years ago due to the civil war. Very soon upon arrival in the host country (the Netherlands), he married a girl from Liberia, also an asylum seeker, and they conceived a daughter.

From early on during their stay in the host country, he committed several violent acts in outbursts of aggression. He had beaten his spouse and fought with police in the street. This is when he was imprisoned for a short period of time, and immediately thereafter admitted at a general psychiatric hospital. While looking at himself in the mirror, Joseph would see his face and body going up in flames. Otherwise, he would see tigers attacking him, but also blood flowing. He would very often hear voices commanding him to kill someone, destroy something, or kill himself because of being a worthless person. Also, from time to time he would hear the voice of his deceased mother calling him to join her. Because of these phenomena, Joseph suffered from high levels of anxiety. Moreover, his mood was depressed, he had suicidal thoughts, he was worried about his life, he suffered from insomnia and fatigue, and he had serious problems concentrating. Joseph had always been physically healthy, with no record of substance abuse.

At the hospital, Joseph was initially diagnosed with a brief psychotic disorder, and then with a severe major depressive disorder with psychotic features, and was treated accordingly. Joseph was first given antipsychotic medication (zuclopentixol 50 mg/day) and antidepressants (sertraline 100 mg/day), but little improvement was reported. Next, the dosage of psychotropic medication was increased, and he became less aggressive and psychotic. The aim of hospitalization was to calm Joseph down and help him with his acute psychiatric symptoms.

Upon release from the hospital, Joseph was referred to an outpatient psychiatric service for further treatment. Joseph was treated by a psychiatrist, and the treatment was, at first, focused on the use of medication. Then, the psychiatrist started applying cognitive-behavioral interventions aimed at controlling the auditive hallucinations that Joseph was suffering from, but the outcomes were not sustainable. At that stage, Joseph was not verbally incoherent any more, and he was able to communicate adequately. The psychiatrist then tried to explore Joseph's cognitions with regard to his depressed mood. However, Joseph showed little interest in talking, and would briefly answer questions. The psychiatrist continued to talk with Joseph primarily about symptoms of his sickness, and not about his past and whereabouts. After some time, Joseph decided to stop seeing his psychiatrist, because he found the sessions meaningless. Soon after he stopped taking medication. After a short period of time, he was re-admitted to a psychiatric ward. This cycle repeated itself a couple of times throughout the next 3 years, until the hospital psychiatrist decided to refer Joseph to a specialized refugee mental health treatment unit for a second opinion and/or continuation of treatment. In the meantime, Joseph's request for asylum was rejected by the immigration authorities. His wife divorced him, and disappeared from his life together with their daughter. At the time of referral to the specialized unit, he was staying illegally in the host country hiding from the police. He slept on the streets, had no money, and would occasionally receive help from charities.

The new therapist assessed Joseph's life history in an in-depth contextual manner. He was actively looking into all issues that are currently accommodated in the CFI (APA, 2013), and gradually, upon witnessing that Joseph could bare it, into traumatic events that Joseph was exposed to in the past. The therapist discovered that Joseph was born into a middle class family in the capital city of Liberia. His parents were Americo-Liberians, an ethnicity whose ancestors were freeborn and formerly enslaved African-Americans who immigrated in the 19th century to become the founders of Liberia. Members of this ethnicity were perceived in Liberia as the most developed and civilized of all ethnic groups. Joseph's father was a lawyer, and his mother a housewife. His father had high expectations with regard to Joseph's upbringing and future. Joseph should one day become a medical doctor. As a child and later as an adolescent, Joseph was expected to get high grades at school. He was not allowed to spend too much time playing with other kids in the street, and every day was brought to school and back home by his father's driver. Joseph described his father's parenting style as firm but fair, and demanding. Joseph was afraid of his father and never dared to contradict him. With his mother, he had a much more affectionate relationship. She would comfort him, and play and talk to him. However, she had to obey her husband, and respect his wishes and plans with regard to their son's upbringing. Upon completing secondary school, Joseph was not able to enroll in a college because of the civil war in Liberia. He would spend most of his time at home, helping his mother in the household. He never had a girlfriend, and he had a couple of friends, all from Americo-Liberian families. Joseph's father had promised to help him financially by opening a car repair workplace as Joseph was interested in mechanics, but the ongoing war forced his father to postpone this plan.

One day, the rebels attacked Joseph's neighborhood and the family home. His parents were first tortured and then killed in front of him; his sister was abducted by the rebels, and he never heard anything about her whereabouts or her destiny. Joseph was severely beaten and tortured, too. Among others, he was forced to chop-off his mother's arm with a machete and lick blood from his father's dead body. The rebels thought that they had killed Joseph. This is why they had left him unconscious, lying in front of the family home next to the bodies of his dead parents. When Joseph regained consciousness, he ran away in panic, leaving his parents’ bodies unburied. A couple of days later, assisted by his parents’ friends, he left the country. He arrived in the Netherlands, and entered a long-lasting procedure of seeking asylum. Joseph had not suffered from psychological complaints of any kind up to the moment that the civil war targeted him.

Although Joseph had a history of torture and war experiences in his home country, he was accused by the immigration authorities of the host country of being a war perpetrator. At the initial interview for asylum, he told his story in an inconsistent way. He had mentioned being a soldier during the war, and having had witnessed a brutal execution of the then president of Liberia. Therefore, 2 years upon arrival in the Netherlands, his asylum request was rejected. Then, he disappeared into illegality in order to avoid repatriation. He led a life without any social rights and in constant fear of being captured by the police. Upon taking his life-story, reading transcripts of his interviews with asylum authorities, and comparing his narrative with available historical documentation, the therapist discovered that asylum authorities had based their decision on wrong data as Joseph was incoherent, confused, and probably psychotic during the interviews for asylum.

First, the therapist assisted Joseph's lawyer and wrote a medical-legal report that was sent to the immigration authorities. They were willing to reconsider their original decision. Joseph was allowed to re-enter the procedure of seeking asylum, return to live in a reception center, and legally await a new decision by the immigration authorities. Next, the psychotic symptoms that Joseph was suffering from started to decrease further.

As the therapist inquired actively into Joseph's life history, he could understand his psychopathology from the perspective of a continuum of posttraumatic reactions. Some of the visual and auditory hallucinations that Joseph was presenting were re-interpreted as flashbacks and re-experiencing of the traumatic events. Joseph would hear voices of the rebel soldiers yelling and humiliating him, and he would re-experience the death of his parents, and see blood flowing. Moreover, he would tell the therapist about being irritable and restless, about having recurring nightmares and flashbacks of the war experiences, about smelling blood, and hearing sounds that were triggering him to remember his violent past.

Second, the therapist tried to engage Joseph with further in-depth exploration of traumatic memories and to start a trauma-focused treatment with imaginal exposure. Joseph refused the therapist's proposal claiming that he was not yet ready for that trajectory. Joseph was afraid of the worsening of his mental health status in case that he would have to talk and think more about his painful past. However, he wanted to continue the treatment and talk about daily problems and existential uncertainties he was facing. Therefore, the therapist focused on supportive interventions. He helped Joseph to identify and avoid triggers for traumatic memories, and structure daily life in the reception center. The second task was not an easy one as there were almost no activities offered at the reception center, no school where Joseph would be able to learn the Dutch language, no opportunity to cook his own meals or to work. In the center, Joseph could only sleep and wait for his asylum request to be processed.

Third, the therapist further examined explanatory models regarding Joseph's mental health complaints. Joseph thought that the flashbacks, re-experiencing, and nightmares were caused by voodoo. He believed they were his punishment, because he had not buried his parents. Their restless, agitated souls were dwelling around and attacking him since they had no place to rest. Joseph, as the only son of his parents, should have taken care of them and not just escaped from the home country. Joseph perceived himself as a coward, a failed child of his parents. The therapist engaged with Joseph in discussions about the meaning of voodoo in the Liberian tribal and the host cultures. This leads to a co-constructed conclusion that the threats from voodoo were not a real danger, but the way social control was exercised in Joseph's native society. Joseph started to put his actions upon the death of the parents into new perspectives, and was beginning to relativize his feelings of shame and guilt with regard to not being able to bury his ancestors. Consequently, anxiety and depressive complaints started to diminish.

Fourth, the therapist discussed with Joseph how to design a ritual for his parents’ burial in the Netherlands. Joseph had made a drawing of his parents, and had chosen to bury it under a tree in a city park. He had created a “grave,” and would go to visit it from time to time. He then started to have new dreams of his parents. In these dreams, they would give him pieces of gold which may help him to rebuild his life. Joseph interpreted the dreams as a sign that his parents were not angry with him anymore, and that they wanted him to live.

Fifth, the therapist discovered that, for Joseph, family was an important resource of strength, and a source of resilience. He assisted him in obtaining a PC, and in enrolling on a computer course, which were both financed by charity. Joseph had heard about possibilities to search his distant relatives in the US through social media. Learning to use a computer gave Joseph a sense of agency, and hope that he may create some links with family members, and diminish the feeling of loneliness.

Sixth, Joseph continued to take psychotropic medication (sertraline 100 mg/day, quetiapine 200 mg/day, topiramate 150 mg/day) and was enabled, only later on, to start with a successful NET (Morina et al., 2012).

Two years later, Joseph still presented with occasional nightmares, flashbacks, and depressed moods, but he was not frozen in fear any more. In sessions with the therapist, he did not complain about these symptoms, but rather talked about existential problems and future outlooks. After many years in the Netherlands, he was still confronted with huge existential uncertainties as his request for asylum was not resolved. However, he had repaired the relationship with his ex-wife and daughter, and was searching for members of his extended family in Liberia and in the United States. He also fell in love with a woman from Kenya, and cherished hope for a better future. Hope emerged in Joseph's life, as he had created some major life-goals. However, we wonder whether all of his symptoms will ever fully disappear? Maybe at the moment that he would obtain a refugee status, and be able to participate fully in the host society, rebuilt his existence, and feel a sense of belonging to a community? Maybe when his wishes to live closer to his daughter, see her on a regular basis, be more involved in her upbringing, and feel wanted and esteemed as a father will be granted? We assume that Joseph will feel better upon feeling safe and attached to others and humanity, experiencing justice in terms of legal recognition of his suffering from human rights abuses, re-taking his identity-roles, and making meaning out of his life, as these are the five core adaptive systems (Silove, 1999) that have been challenged by his violent past.

## Comments on the case presentation—a joy of discovering the obvious?

We argue that Joseph suffered, from the very beginning of his treatment, from PTSD due to exposure to multiple traumatic experiences. However, throughout time he presented with a spectrum of different reactions to trauma exposure. At the hospital admission, acute psychotic and associated mood disorder features were the most prominent, but there were symptoms of PTSD, too. As the treatment unfolded, medication helped diminish the psychotic, and the depressive symptoms to a certain extent, but the clinician failed to explore Joseph's life history, and gain insight into his traumatic past. The clinician may have been fearful of further destabilizing Joseph by asking questions about the painful past events. Or he was not knowledgeable with regard to the continuum of PTSD reactions, and the possibility that psychosis, depression, and PTSD may be linked conditions caused by the same underlying mechanism. Therefore, only later on in the course of his treatment, a contextual, in-depth analysis of Joseph's life trajectory helped to understand his psychopathology from a different perspective, and shaping a set of treatment interventions.

We argue that Joseph's proneness to psychosis, as a reaction to exposure to extreme violence, has been augmented by a set of both developmental and contextual factors. First, the traumatic events that Joseph was exposed to were of an extremely violent nature, and may have overwhelmed his coping mechanisms. Further, it may be that Joseph was insufficiently separated and individuated from his parents, and brought up in a very protected way. Therefore, he may have experienced great difficulties with his parents’ violent deaths, and in reattaching to others in an unfamiliar environment of the host country. Moreover, upon forced migration Joseph had lost all resources (personal, material, and conditional), and he had received no opportunities to rebuild them in the host society. This may have augmented his sensitivity for war-related stress. Further, multiple resettlement stressors had accumulated throughout the years, and may have strongly challenged Joseph's resilience capacities. A need for existential safety and a goal to rebuild his life were also aggravated throughout his stay in exile.

Due to a presence of problems on multiple levels in Joseph's ecological environment, the treatment was a combination of interventions targeting intrapsychic (multimodal, and only later on, trauma-focused psychotherapy, psychotropic medication), interpersonal (re-bonding with humanity through the therapeutic alliance), resilience (learning about computers), and environmental issues (advocacy activities targeting resettlement stress) in a culture-sensitive way (explanatory models, voodoo, ritual burial). Moreover, Joseph was assisted in regaining a sense of control over his own life to a certain extent, and in restoring hope regarding the future. We argue that the timing and sequence of the applied interventions were important for successful treatment. The treatment strategy was co-constructed with the patient taking into account his hierarchy of needs. Only after assisting Joseph in the context of ongoing threat, he was willing and able to confront traumatic memories and tolerate elevated anxiety. However, while successfully engaging with the trauma-focused treatment later on, he still had to cope with many existential uncertainties, suggesting that the application of trauma-focused techniques did not result in adverse emotional reactions and worsening of Joseph's mental health condition. We argue that the treatment was a balancing act between addressing the concerns that are having the most significant impact on Joseph's life and alleviating trauma-related distress in a way that facilitated functional improvement. The applied multimodal interventions may have diminished psychosocial distress, improved Joseph's functioning, increased motivation for trauma-focused therapy, and helped avoid dropout from the treatment, as the treatment unfolded in a way that was meaningful to the patient.

We suggest that in cases of refugees with severe PTSD and the invalidating presence of current psychosocial stress, clinicians should aim at collecting information that will enhance understanding of the development of mental health problems across a patient's lifespan and in the ecological environment. They should be led by a causality principle in development of psychiatric problems and question why a patient has developed psychopathology at a certain moment in his/her life, what has protected him/her from becoming sick earlier in life and which, previously present, sources of resilience may be strengthened in order to help a patient to get better. The Integrative Contextual Model (Drožđek, 2007; Drožđek et al., 2012a, 2012b) may help them to gain the insights needed.

Further, the CFI (APA, 2013) can be used as a comprehensive assessment tool which inquires into the cultural definitions of problems that patients are presenting with, cultural perceptions of cause, context, and support received from family, friends or community, the role of cultural identity, cultural factors affecting self-coping, present and past help seeking, preferences for, and barriers to care, as well as clinician-patient relationship. This tool also provides information regarding explanatory models of sickness, illness prototypes, expectations from treatment, level of functioning, impact of an internal social network on illness, psychosocial stressors, spirituality, religion, and moral traditions, and is designed for use with every patient in clinical practice.

Although applying the Integrative Contextual Model (Drožđek, 2007; Drožđek et al., 2012a, 2012b) may be time consuming, we argue that it may improve: (1) assessment of patients’ problems, (2) design and timing of interventions on different levels of the ecological environment, and (3) evaluation thereof. However, we argue that interventions in the domains of psychology and psychiatry should, where available, be methodologically sound, and based on scientific evidence. In other words, we expect that the initial time investment will pay off later on in the course of treatment of severely traumatized refugees, as adequately tailored treatment strategies may produce better and more sustainable outcomes.

Empirical and clinical knowledge about which combinations of interventions may be effective and which may not should be studied. Moreover, future research should examine outcomes of the evidence-based, and other interventions within the context on both, PTSD symptoms, and issues like quality of life, resilience, social integration, or social relationships.

## Concluding remarks

Multimodal and trauma-focused interventions can be combined in the treatment of refugees with severe PTSD and comorbidity. The Integrative Contextual Model (Drožđek, 2007; Drožđek et al., 2012a, 2012b) for the understanding of mental health disorders may help with shaping the treatment strategy.

The recently launched DSM-5 (APA, 2013) is more inclusive with regard to the cultural and contextual determinants of psychiatric disorders than any previous diagnostic manual. Therefore, we hope that a momentum has come for clinicians to consider more comprehensive practices in the treatment of PTSD in order to assist their severely traumatized refugee patients more successfully.

## Supplementary Material

Challenges in treatment of posttraumatic stress disorder in refugees: towards integration of evidence-based treatments with contextual and culture-sensitive perspectives

Challenges in treatment of posttraumatic stress disorder in refugees: towards integration of evidence-based treatments with contextual and culture-sensitive perspectives

Challenges in treatment of posttraumatic stress disorder in refugees: towards integration of evidence-based treatments with contextual and culture-sensitive perspectives

Challenges in treatment of posttraumatic stress disorder in refugees: towards integration of evidence-based treatments with contextual and culture-sensitive perspectives

Challenges in treatment of posttraumatic stress disorder in refugees: towards integration of evidence-based treatments with contextual and culture-sensitive perspectives
