# Supplementary material for: Challenges in treatment of posttraumatic stress disorder in refugees: towards integration of evidence-based treatments with contextual and culture-sensitive perspectives
Source: Eur J Psychotraumatol. 2015 Jan 7;6:10.3402/ejpt.v6.24750. doi: 10.3402/ejpt.v6.24750 (PMC4287632; doi:10.3402/ejpt.v6.24750)
Supplement: Challenges in treatment of posttraumatic stress disorder in refugees: towards integration of evidence-based treatments with contextual and culture-sensitive perspectives [file EJPT-6-24750-s001.pdf]

Challenges in treatment of post traumatic stress disorder in refugees: Towards integration of evidence-based treatments with contextual and culture-sensitive perspectives

Boris Drožđek

Pozadina:
U tretmanu izbjeglica s kompleksnim posttraumatskim stresnim poremećajem (PTSP) danas se najčešće koriste psihološke intervencije čiji je fokus konfrontacija s traumatskim sjećanjima te složene, tzv. multimodularne intervencije koje su kombinacija različitih terapijskih pristupa. Dok za efikasnost prvih postoje preliminarni znanstveni dokazi, kod multimodularnih intervencija to još nije slučaj. Moguće je da su multimodularne intervencije bile istraživane u populacijama koje su otpornije na psihološki tretman i koje pate od ozbiljnijih psiholoških poremećaja, kronične psihopatologije te teže razine invaliditeta. Usprkos pomanjkanju znanstvenih dokaza, multimodularne intervencije se uveliko primjenjuju u liječenju izbjeglica sa PTSP-om. Stručnjaci smatraju da se adekvatna pomoć ovoj populaciji sastoji od intervencija kojima je cilj unaprijediti psihološko zdravlje, kako smanjivanjem simptoma psihopatologije, tako i utjecanjem na psihosocijalne stresove kojima obiluje svakodnevni život izbjeglica.
U proteklih nekoliko desetljeća razvijeni su različiti modeli za bolje razumijevanje složenih odnosa između psiholoških problema i zdravlja. Ti modeli pokušavaju spoznati i objasniti psihološke poremećaje, vodeći računa o jedinstvenim kontekstualnim determinantama pojedinca te bi mogli biti od koristi u procesu dizajniranja učinkovitih multimodularnih intervencija.
Cilj:
Usmjeriti pažnju terapeuta na mogućnost korištenja Integrativnog kontekstualnog modela u procesu razumijevanja i dijagnosticiranja kompleksnih posttraumatskih mentalnih poremećaja. Ovaj model može biti od koristi i kod planiranja psihološkog/psihoterapijskog tretmana koji uključuje intervencije čiji je fokus konfrontacija s traumatskim sjećanjima u multimodularni tretman. Cilj ove kombinacije tretmana je poboljšanje psihološke pomoći izbjeglicama s PTSP-om.
Metoda:
Korištenje Integrativnog kontekstualnog modela u tretmanu teškog oblika PTSP-a kod izbjeglica opisano je i analizirano na osnovi znanstvene literature, kliničkog iskustva i prezentacije fiktivnog slučaja.
Rezultati:
Integrativni kontekstualni model, kojim se produbljuje znanje o kontekstualnim i razvojnim faktorima koji bi mogli utjecati na posttraumatsku psihopatologiju, pomaže razumijevanju kompleksnih psiholoških oštećenja kao posljedica teške traume. Ovaj model može biti od koristi i pri dizajniranju kombiniranog pristupa liječenju PTSP-a kod izbjeglica koji uključuje i psihološke intervencije usmjerene na konfrontaciju s traumatskim sjećanjima.
Zaključak:
Pružanje psihološke pomoći traumatiziranim izbjeglicama moglo bi biti unaprijeđeno upotrebom modela za bolje razumijevanje složenih posttraumatskih reakcija. Prikazani Integrativni kontekstualni model se zasniva na kontekstualnim i razvojno-psihološkim pristupima mentalnom zdravlju i bolesti. Multimodularne intervencije i one usmjerene na konfrontaciju s traumatskim sjećanjima mogu se kombinirati u tretmanu izbjeglica sa složenim oblikom PTSP-a.

Citation: European Journal of Psychotraumatology 2014, **6**: 24750 - http://dx.doi.org/10.3402/ejpt.v6.24750
