# Supplementary material for: Challenges in treatment of posttraumatic stress disorder in refugees: towards integration of evidence-based treatments with contextual and culture-sensitive perspectives
Source: Eur J Psychotraumatol. 2015 Jan 7;6:10.3402/ejpt.v6.24750. doi: 10.3402/ejpt.v6.24750 (PMC4287632; doi:10.3402/ejpt.v6.24750)
Supplement: Challenges in treatment of posttraumatic stress disorder in refugees: towards integration of evidence-based treatments with contextual and culture-sensitive perspectives [file EJPT-6-24750-s002.pdf]

**Challenges in treatment of post traumatic stress disorder in refugees: Towards integration of evidence-based treatments with contextual and culture-sensitive perspectives**

**Boris Drožđek**

Achtergronden:
Wetenschappelijk onderzoek suggereert dat trauma-focus therapieën en multimodale behandelingen de meest gebruikte strategieën zijn in de hulpverlening aan asielzoekers en vluchtelingen met een posttraumatische stress stoornis (PTSS). Terwijl de effectiviteit van de trauma-focus therapieën bewezen is, zijn er slechts voorlopige wetenschappelijke bewijzen voor effectiviteit van de multimodale interventies. Deze interventies worden, vaak ingezet vanuit de redenatie dat deze doelgroepen t, (de asielzoekers en de vluchtelingen,) naast de intrapsychische beschadigingen ook kampen met vele actuele psychosociale stressoren. De behandeling moet daarom een combinatie van diverse aanpakken met verschillende doelgebieden zijn.
De effectiviteit van multimodale behandelingen is wellicht slechts marginaal aangetoond omdat deze interventies onderzocht zijn in meer therapieresistente populaties met ernstigere, chronischere en meer invaliderende psychopathologie.

In de afgelopen decennia zijn erschillende modellen voor het ontrafelen van de complexe relatie tussen de geestelijke gezondheid en ziekte ontworpen. Hun doel was om de geestelijke gezondheidsproblemen te benaderen en te begrijpen vanuit de geïndividualiseerde, contextuele, epigenetische en cultuursensitieve perspectieven.

Doel:
De aandacht van clinici te vestigen op de waarde van het gebruik van het Integratief contextueel model in de dagelijkse klinische praktijk met getraumatiseerde asielzoekers en vluchtelingen. Dit model integreert de contextuele en de ontwikkelingsparadigmas' en impliceert het kaderen en interpreteren van menselijke ervaringen en de gevolgen daarvan in "de ecologische omgeving". Het brengt de effecten van traumatische ervaringen op verschillende niveaus van het menselijk bestaan in kaart en schept mogelijkheden voor behandelaren tot het ontwerpen en evalueren van toegesneden behandelingsstrategieën. De hulpverlener krijgt bij toepassing van dit model een beter inzicht in de bronnen van veerkracht bij overlevenden van trauma. Als gevolg daarvan omvat een adequate behandelingsstrategie ook interventies buiten de domeinen van de medische en de psychologische behandelingen en is gericht op zowel bronnen van veerkracht als posttraumatische schade op de verschillende niveaus van de 'ecologische omgeving' van de overlevende.

Methode:
Het gebruik van het Integratief contextueel model in behandeling van complexe PTSS bij een vluchteling wordt gepresenteerd en gediscuteerd op basis van wetenschappelijke literatuur, klinische ervaring en presentatie van een casus.

Resultaten:
Het Integratief contextueel model kan helpen met het ontwerpen van multimodale interventies en het combineren van deze interventies met de trauma-focus therapieën in behandeling van asielzoekers en vluchtelingen met een PTSS.

Samenvatting:

Hulpverlening aan asielzoekers en vluchtelingen met complexe posttraumatische beschadigingen kan verfijnt worden met het gebruik van het Integratief contextueel model voor het begrijpen en in kaart brengen van complexe psychologische en psychosociale gevolgen van blootstelling aan trauma. Trauma-focus interventies kunnen onderdeel maken van een multimodale behandeling van PTSS.

**Citation:** European Journal of Psychotraumatology 2014, **6**: 24750 - http://dx.doi.org/10.3402/ejpt.v6.24750
