# Supplementary material for: Challenges in treatment of posttraumatic stress disorder in refugees: towards integration of evidence-based treatments with contextual and culture-sensitive perspectives
Source: Eur J Psychotraumatol. 2015 Jan 7;6:10.3402/ejpt.v6.24750. doi: 10.3402/ejpt.v6.24750 (PMC4287632; doi:10.3402/ejpt.v6.24750)
Supplement: Challenges in treatment of posttraumatic stress disorder in refugees: towards integration of evidence-based treatments with contextual and culture-sensitive perspectives [file EJPT-6-24750-s003.pdf]

Herausforderungen bei der Behandlung von posttraumatischen Belastungsstörungen bei Flüchtlingen: Integration von evidenzbasierter Behandlung mit kontextueller und kultursensibler Perspektive

Boris Drozdek

Hintergrund: Die Forschung zeigt, dass Trauma-Therapie und multimodale Interventionen die beiden meist verwendeten Strategien im Umgang mit Flüchtlingen sind, die an einer posttraumatischen Belastungsstörung (PTBS) leiden. Während erste Hinweise darauf deuten, dass auf Traumata konzentrierte Ansätze eine gewisse Wirksamkeit zeigen, konnte dies nicht für multimodale Interventionen bestätigt werden. Allerdings kann es sein, dass multimodale Interventionen eher bei „behandlungsresistenteren“ Flüchtlingen untersucht wurden, welche ein höheres Level an Psychopathologie, Behinderung und Chronizität aufweisen. In den vergangenen Jahren sind verschiedene Modelle entstanden, um das komplexe Verhältnis zwischen psychischen Problemen und Wohlbefinden zu verstehen. Sie zielen darauf ab, psychische Probleme auf die individualisierten, kontextabhängigen, epigenetischen und kulturell empfindlichen Weisen zu gestalten und sind möglicherweise bei dem Erstellen des Inhalts und der zeitlichen Regelung von multimodalen Interventionen nützlich.

Ziel: Ziel dieser Studie war es, die Aufmerksamkeit der KlinikerInnen auf die Möglichkeit der Anwendung des integrativen kontextabhängigen Modells zu lenken. Außerdem sollte auf Verständnis und Einschätzung der Folgeerscheinungen von Traumata auf psychische Gesundheit beim Herstellen von multimodalen Interventionen fokussiert werden, um eine Kombination multimodaler evidenzbasierter Ansätze darzustellen, die das Verständnis und die Behandlung von PTBS und anderen psychischen Problemen der Überlebenden eines längeren und sich wiederholenden Traumas verbessern können.

Methode: Basierend auf Literatur, klinischer Erfahrung und der Präsentation eines Falls wird der Gebrauch des einheitlichen Kontextmodells in der Erstellung des Behandlungsplanes von schwerer PTBS bei einer FlüchtlingspatientIn präsentiert und besprochen.

Ergebnisse: Das integrative kontextuelle Modell für Verständnis und Bewertung von Faktoren, die eine Rolle bei der Entstehung und Aufrechterhaltung von PTBS und Komorbidität bei Flüchtlingen spielen, kann helfen, maßgeschneiderte multimodale Interventionen zu erstellen. Diese Interventionen können mit evidenzbasierter Trauma-orientierter Behandlung kombiniert werden.

Schlussfolgerung: Das Themengebiet der psychischen Gesundheit von Flüchtlingen und der Interventionen in der klinischen Praxis mit traumatisierten Flüchtlingen kann mit Modellen zur Bewertung und Konzeption von Trauma-Folgen gut ergänzt werden. Multimodale und Trauma-fokussierte Interventionen können sequenziell in einen Behandlungsverlauf aufgenommen werden.

Keywords/Schlagwörter: Flüchtlinge, PTBS, Intervention, Therapie, Kontext, Erarbeitung kultureller Interviews, organisierte Gewalt, Kultur

Name of translator: Iris Fischer, Brigitte Lueger-Schuster

Citation: European Journal of Psychotraumatology 2015, 6: 24750 - http://dx.doi.org/10.3402/ejpt.v6.24750
