# Supplementary material for: Challenges in treatment of posttraumatic stress disorder in refugees: towards integration of evidence-based treatments with contextual and culture-sensitive perspectives
Source: Eur J Psychotraumatol. 2015 Jan 7;6:10.3402/ejpt.v6.24750. doi: 10.3402/ejpt.v6.24750 (PMC4287632; doi:10.3402/ejpt.v6.24750)
Supplement: Challenges in treatment of posttraumatic stress disorder in refugees: towards integration of evidence-based treatments with contextual and culture-sensitive perspectives [file EJPT-6-24750-s004.pdf]

Boris Drozdek

Abstract
Zmiany w leczeniu zaburzenia po stresie traumatycznym wśród uchodźców: w kierunku integracji terapii opartych na danych naukowych z kontekstualno-kulturowym podejściem terapeutycznym
Wprowadzenie: Badania wskazują, że terapia skoncentrowana na traumie oraz interwencje multimodalne są dwoma najczęściej stosowanymi metodami leczenia zaburzenia po stresie traumatycznym wśród uchodźców. Jeśli badania empiryczne dostarczają danych na pewien stopień skuteczności terapii skoncetrowanej na traumie w leczeniu PTSD wśród tej grupy osób, to jednak tego samego nie można powiedzieć o interwencjach multimodalnych. Być może jednak jest to spowodowane tym, że interwencje multimodalne były stosowane wśród pacjentów z wyższym nasielniem objawów psychopatologicznych. Być może koniecznym jest uzupełnienie dotychczasowych metod leczenia o podejście kontekstualno-kulturowe.
Cel: Celem niniejszej pracy było zwrócenie uwagi klinicystów na przydatność Zintegrowanego Modelu Kontekstualnego (ang. the Integrative Contextual Model) przy zrozumieniu i pomiarze objawów PTSD wśród uchodźców. Niniejsze parca dązy też do wzbogacenia obecnie istniejącyhc metod leczenia PTSD.
Metoda: Bazując na literaturze przedmiotu, doświadczeniu klinicznym oraz prezentacji fikcyjnych studiów przypadku, dyskutowana jest przydatność Zintegrowanego Modelu Kontekstualnego w leczeniu PTSD wśród uchodźców.
Wyniki: Zintegrowany Model Kontekstualny może być wykorzystany w zrozumieniu w powstawaniu i utrzymywaniu się PTSD wśród uchodźców. Model ten może być połączyny z terapiami PTSD opartymi na danych naukowych.
Konkluzje: Problemtyka zdrowia psychciznego wsród uchodźców nie jest do końca zbadane. Użycie Zintegrowanego Modelu Kontekstualnego może znacznie rozjaśnić tą tematyke.
Słowa kluczowe: uchodźcy; PTSD; interwencja; terapia; kontekst; wywiady kulturowe; zorganizowana przemoc; kultura.

Name of translator: Marcin Rzeszutek, University of Finance and Management in Warsaw, Poland

Citation: European Journal of Psychotraumatology 2015, 6: 24750 - http://dx.doi.org/10.3402/ejpt.v6.24750
