# Supplementary material for: Challenges in treatment of posttraumatic stress disorder in refugees: towards integration of evidence-based treatments with contextual and culture-sensitive perspectives
Source: Eur J Psychotraumatol. 2015 Jan 7;6:10.3402/ejpt.v6.24750. doi: 10.3402/ejpt.v6.24750 (PMC4287632; doi:10.3402/ejpt.v6.24750)
Supplement: Challenges in treatment of posttraumatic stress disorder in refugees: towards integration of evidence-based treatments with contextual and culture-sensitive perspectives [file EJPT-6-24750-s005.pdf]

Mültecilerde Travma sonrası stres bozukluğu tedavisindeki zorluklar: Kanıta dayalı tedavilerin bağlamsal ve kültüre duyarlı perspektifler ile bütünleştirilmesi yolunda

Boris Drozdek

Özet
Arkaplan: Araştırmalar, travma odaklı terapi ve multimodal müdahalelerin travma sonrası stres bozukluğundan (TSSB) muzdarip mültecilerin tedavisinde en sık kullanılan iki strateji olduğunu göstermektedir. Ön kanıtlar travma odaklı yaklaşımların belli bir etkinliği olabileceğini gösterirken, aynısı multimodal müdahaleler için tespit edilememiştir. Ancak bu, multimodal müdahalelerin tedaviye daha dirençli, çok yüksek psikopatoloji seviyeleri, engellilik hali ve kronisitesi olan mülteciler üzerinde çalışılmasından kaynaklanabilir.
Geçtiğimiz onlarca yıl boyunca, ruh sağlığı sorunları ve iyi oluş arasındaki karmaşık ilişkiyi anlamak adına çeşitli modeller ortaya çıkmıştır. Ruh sağlığı sorunlarının kişisel, bağlamsal, epigenetik ve kültüre duyarlı yollarla çerçevelendirilmesi multimodal müdahalelerin içeriği ve zamanlamasını değiştirmede faydalı olabilir.
Amaç: Multimodal ve kanıta dayalı travma odaklı yaklaşımların birleşiminin olası bir yolunu sunmak ve uzun sureli ve süregelen travma geçirmiş mültecilerin TSSB ve diğer ruh sağlığı sorunlarının anlaşılması ve tedavilerinin iyileştirmesi amacıyla Multimodal müdahaleleri değiştirerek travma sonrası ruh sağlığı sekellerini anlama ve ölçmede Entegre Bağlamsal Modelin kullanılma olasılığına klinisyenlerin dikkatini çekmek.
Yöntem: Literatüre, klinik deneyim ve kurgusal bir vakaya dayanarak, ciddi TSSB'si olan bir mültecinin tedavisinin değiştirilmesinde Entegre Bağlamsal Modelin kullanımı sunulmuş ve tartışılmıştır.
Sonuçlar: Mültecilerde TSSB'nin başlangıcı, devamı ve eşlik etmesinde rol oynayan etmenleri anlama ve ölçmede Entegre Bağlamsal Modelin kullanımı multimodal müdahalelerin değiştirilmesinde faydalı olabilir.
Tartışma: Mülteci ruh sağlığı müdahaleleri ve travmatize olmuş mültecilerdeki klinik uygulama alanları, travma sonrası sekellerin ölçülmesi ve kavramsallaştırılmasında bağlamsal ve gelişimsel modellerin kullanılmasıyla zenginleştirilebilir. Tedavinin gidişat sürecinde multimodal ve travma odaklı müdahaleler sırayla uygulanabilir.

Anahtar kelimeler: mülteciler; TSSB; müdahale, terapi; bağlam; kültür formasyonu görüşmesi; organize şiddet; kültür.

Name of translator: Emek Yuce Zeyrek-Rios

Citation: European Journal of Psychotraumatology 2015, 6: 24750 - http://dx.doi.org/10.3402/ejpt.v6.24750
